# Social inequalities in cervical cancer screening: a discrete choice experiment among French general practitioners and gynaecologists

**DOI:** 10.1186/s12913-020-05479-w

**Published:** 2020-07-27

**Authors:** Thibaut Raginel, Guillaume Grandazzi, Guy Launoy, Mélanie Trocmé, Véronique Christophe, Célia Berchi, Lydia Guittet

**Affiliations:** 1 NORMANDIE UNIV, UNICAEN, INSERM, ANTICIPE, 14000 Caen, France; 2 NORMANDIE UNIV, UNICAEN, UFR Sante, Department of General Practice, 14000 Caen, France; 3 Univ. Lille, UMR 9193 - SCALab - Sciences Cognitives et Sciences Affectives, F-59000 Lille, France; 4 Caen University Hospital, Département d’Information Médicale, Avenue de la Côte de Nacre, 14033 Caen, France

**Keywords:** Early detection of cancer, Choice behavior, General practitioners, Healthcare disparities, Uterine cervical neoplasms, Primary health care

## Abstract

**Background:**

Cervical cancer screening is effective in reducing mortality due to uterine cervical cancer (UCC). However, inequalities in participation in UCC screening exist, especially according to age and social status. Considering the current situation in France regarding the ongoing organized UCC screening campaign, we aimed to assess general practitioners’ (GPs) and gynaecologists’ preferences for actions designed to reduce screening inequalities.

**Methods:**

French physicians’ preferences to UCC screening modalities was assessed using a discrete choice experiment. A national cross-sectional questionnaire was sent between September and October 2014 to 500 randomly selected physicians, and numerically to all targeted physicians working in the French region Midi-Pyrénées. Practitioners were offered 11 binary choices of organized screening scenarios in order to reduce inequalities in UCC screening participation. Each scenario was based on five attributes corresponding to five ways to enhance participation in UCC screening while reducing screening inequalities.

**Results:**

Among the 123 respondents included, practitioners voted for additional interventions targeting non-screened women overall (*p* <  0.05), including centralized invitations sent from a central authority and involving the mentioned attending physician, or providing attending physicians with the lists of unscreened women among their patients. However, they rejected the specific targeting of women over 50 years old (*p* <  0.01) or living in deprived areas (*p* <  0.05). Only GPs were in favour of allowing nurses to perform Pap smears, but both GPs and gynaecologists rejected self-collected oncogenic papillomavirus testing.

**Conclusions:**

French practitioners tended to value the traditional principle of universalism. As well as rejecting self-collected oncogenic papillomavirus testing, their reluctance to support the principle of proportionate universalism relying on additional interventions addressing differences in socioeconomic status needs further evaluation. As these two concepts have already been recommended as secondary development leads for the French national organized screening campaign currently being implemented, the adherence of practitioners and the adaptation of these concepts are necessary conditions for reducing inequalities in health care.

## Background

In Europe, uterine cervical cancer (UCC) was the fifth most frequent cancer for incidence and the seventh for mortality in women with roughly 58,000 annual new cases and 24,000 annual deaths in 2012 [1]. The incidence varies across Europe, with a higher age-standardised incidence rate in countries in Central and Eastern Europe than in Western Europe countries such as France. It is now demonstrated that regular screening with Pap smear is effective in reducing mortality [2]. The implementation of organized screening campaigns in some countries has been a determining factor in the favourable epidemiological evolution of the disease, and most European countries have adopted recommendations in this regard [3]. Nevertheless, the modality of UCC screening differs between countries, not all countries in Europe having yet established a national organized screening programme [4]. In addition, specific vaccines have been developed against oncogenic human papillomavirus (HPV), a major cause of UCC. The arsenal of preventive tools against UCC is complemented by HPV screening techniques that identify women at risk of intraepithelial lesions (triage test for UCC screening) [5]. Some HPV screening techniques rely on self-sampling that may help women reluctant to undergo a gynaecological examination to benefit from pre-screening. Several studies have demonstrated that HPV self-sampling screening may be highly acceptable with good uptake [6, 7], and are cost-effective [8]. Therefore, the morbimortality associated with UCC is highly preventable in countries with a modern and effective health care system.

Social inequalities are observed in the field of UCC prevention and the underlying mechanisms may be varied [9–11]. The latter include individual factors such as perceived risk of UCC and its consequences, reluctance to undergo a gynaecological examination, lack of regular medical follow-up, cost of Pap smears and subsequent diagnostic explorations in the event of a positive test [9, 12]. In addition, several contextual factors are also involved including heterogeneity of the geographical density of gynaecologists, general practitioners (GPs) or midwives, which affect access to Pap smear operators and prescribers [9, 12]. Such social inequalities in screening are not specific to UCC and are observed even in organized screening programmes with free screening [13–16]. However, organized screening programmes can reduce UCC screening inequalities [17], especially when specific measures [18] are used to target deprived populations. The support of primary care practitioners is essential for the success of an organized screening programme, especially those involved in gynaecological follow-up [12]. However, little is known about acceptability of interventions which could limit inequalities in participation to UCC screening from the practitioners’ point of view.

The aim of this article is to present the evolution of cervical cancer screening in France, with a focus on a discrete choice experiment conducted among general practitioners and gynaecologists in order to prevent inequalities occurring within the ongoing national organized UCC screening campaign. The discrete choice experiment was chosen to assess the acceptability of practitioners with regard to the possible interventions.

### History of cervical cancer screening in France

In France, the first official recommendations for UCC screening were published in 1990 [19] as individual opportunistic screening. Women aged from 25 to 65 years old are recommended to undergo individual opportunistic cytological UCC screening every 3 years after two normal cytological UCC screenings 1 year apart [9]. In 2002 the French agency in charge of health care guidelines (ANAES) specified that the investigation should be conducted in the event of an abnormal Pap smear [20]. At this period, Pap smear was proposed by practitioners without any systematic invitation.

Between 1990 and 2010, four French departments began local organized UCC screening programmes based on invitations sent either to all women aged 25 to 65 years old, or only to those not having performed any Pap smear in the last 3 years, and based on lists produced by health insurance schemes (HIS). The cost of Pap smear analysis only was fully paid by a third party (paid directly by the HIS) in half of the programs. In 2010, these pilot UCC screening programs decided on a common policy of inviting women only 25–65 years old not having performed any Pap smear in the last 3 years, with a reminder 1 year later if necessary. Nine extra departments then joined the experimentation. This standardized program offered a 10% increase in participation in UCC screening [21]. However, this experimentation in UCC screening was not implemented throughout France, unlike the campaigns for breast cancer (first programs in 1989 were extended nationwide in 2004) [22] and for colorectal cancer (the program begun in 2002 was extended nationwide in 2008) [23]. All programs involved local screening organizations. The decision to roll out UCC screening nationwide was taken in 2018, and its actual realization is expected at the end of 2019. Women with no Pap smear in the last 3 years will be invited to undergo one free of charge.

### Organization of the French health care system

The French population is covered by national Health Insurance Schemes and medical care is reimbursed. There is a gatekeeper system. However, a) the gatekeeper should be a physician but not necessarily a general practitioner; b) a patient may consult outside the system with reduced but not zero reimbursement; c) access to gynaecologists is not subjected to the gatekeeper process. The gynaecological follow-up of women is performed by GPs and gynaecologists. The involvement of midwives was previously restricted to pregnancy but is now allowed outside pregnancy. Therefore, Pap smear screening may be done by GPs, gynaecologists, midwives or laboratory analysis centres following the prescription of one of the abovementioned professionals.

Apart from salaried doctors in public or private hospitals or clinics, health care centres, etc., French practitioners have been paid historically on a fee-for-service basis with a fixed fee for each act performed themselves or under their supervision. For example, for each Pap smear, the collecting practitioner may charge the code JKHD001 of € 12.46 in addition to the fee for the consultation. All fees are reimbursed to women on the same basis. This remuneration system has recently been diversified with the introduction of complementary partial remunerations: a) a remuneration for capitation, i.e. a lump sum per individual whose doctor is the declared attending physician, without considering the number of acts performed for these individuals each year; and b) a payment for performance system, also called “ public health objectives” in the French terminology [24]. The latter grants each doctor an annual number of points obtained for meeting several mutually agreed objectives. Each point has a defined monetary value and the total remuneration is weighted on various criteria including the number of patients whose doctor is declared the attending physician, the percentage of deprived patients receiving free supplementary universal health coverage in a practitioner’s care, etc. In 2011, the objective concerning Pap smear coverage was a target rate of 80% of women aged 25 to 65 years old whose doctor was declared the attending physician in the previous 3 years. The current convention signed in 2016 has lowered this target to an intermediate objective of 52% and a final target of 65%. Reaching this final target is rewarded with the 40-point maximum number of points for that item. In 2019, each point was worth 7 euro.

Two national organized cancer screening programs are implemented for breast and colorectal cancer (CRC). In these programs, the lists of individuals aged 50–74 are transmitted by the HIS to the local organizations in charge of the systematic biennial invitations, of the follow-up of tests including second reading of mammography, and of collecting results of confirmatory tests (respectively biopsies or colonoscopies) in the event of a positive mammography or faecal occult blood test (FOBT). Mammography should be performed in specialized quality-certified centres. FOBTs are distributed by GPs during regular consultations since there are no specific reimbursed consultations dedicated to screening. The mammography and the FOBTs are free of charge, but confirmatory exams in the event of a positive test are reimbursed by the HIS as in other situations (70 to 100% of cost reimbursed according to diagnostic process). The mammography can also be performed outside the national organized screening program. Although not recommended in the absence of risk factors, colonoscopy screening can be prescribed on a discretionary basis and, if so, is reimbursed 70 to 80%. Both screening programs are based on the principle of equality, every woman receiving the same care. However, social inequalities in participation have been demonstrated in both of these organized screening programs [11, 25–28].

Nationwide rollout of UCC screening will be slightly different in some respects. Considering opportunistic screening, neither Pap smear screening nor diagnostic explorations following a positive test have been fully reimbursed until now (70 to 80% reimbursement). As in breast cancer and CRC screening programs, the national UCC screening program will provide a free-of-charge analysis of Pap smears. However, Pap smear sampling, associate consultations, and confirmatory exams in the event of a positive test will remain reimbursed, as in other situations (70 to 80% reimbursement). The UCC programme will also differ in other regards. First, the invitation for UCC screening will be sent only to women not participating spontaneously, whereas generalized to the whole age-group population for breast and colorectal cancer screening. Second, Pap smears will still have to be taken by a gynaecologist, GP, medical staff in a laboratory or a midwife.

The next section presents the results of a cross-sectional questionnaire-based survey conducted before the publication of the official French guidelines on UCC screening, whose results could guide adaptation of the programme in the future.

### Discrete choice experiment

#### Methods

A Discrete Choice Experiment (DCE) [29] was conducted in 2014 among a sample of French GPs and gynaecologists. Briefly, the study consisted of a self-administered questionnaire presenting practitioners with a set of 11 pairs of hypothetical scenarios for UCC screening (see blank English language copy of questionnaire in additional file). For each pair, they were asked to choose the one they preferred in order to reduce inequalities in participation in UCC screening.

The attributes and modalities for developing the scenarios were based on a preliminary qualitative study (not detailed in this paper) based on semi-structured interviews of several stakeholders in the health care systems involved in cancer screening, complemented by a literature review of physicians’ representations of health inequalities in cancer screening, and interventions for reducing social inequalities in cancer screening [3, 6, 9, 12, 17, 18, 30–36].

Table 1 shows the modalities used for the selected scenarios with five attributes. Each attribute included a ‘neutral’ modality reflecting usual non-organized opportunistic UCC screening in France (first line of each attribute in Table 1). In each scenario, the first attribute specified the population targeted by the scenario, whilst the modalities of all the other attributes were intended to be applied to this population. For women not targeted by the scenario, UCC screening was supposed to follow the usual non-organized opportunistic programme in France. The OPTEX procedure in the SAS 9.2 software was used to reduce the number of scenarios to be proposed, leading to 22 scenarios allocated in 11 pairwise choices. After the OPTEX procedure, the produced scenarios were checked and validated by several revisions. First of all, the research team analysed all regrouped modalities used in each scenario in order to check whether the scenarios produced were workable if implemented and consistent. Secondly, the scenarios produced were tested on two samples of practitioners: the first sample of 11 practitioners for the postal survey and the second of nine practitioners for the online survey. None of the revisions of the questionnaire required any modification within the scenarios apart from the spelling corrections.

**Table 1:** Attributes and modalities included in scenarios according to qualitative interviews and literature

Population of women targeted	Stakeholders in screening itself	UCC screening technique(s)	Inducement to women to undergo screening	Inducement to general practitioners
All women ^a^	Current stakeholders ^a^	Pap smear ^a^	Current incentives for screening ^a^	No change in remuneration or logistic support ^a^
Unscreened women	Current stakeholders and state-registered nurses	Choice between Pap smear or self-collected oncogenic papillomavirus testing	Mailed invitation without involving attending physician	Increasing fee for performing Pap smear
Women from areas with low rates of screening	Current stakeholders and radiologists during mammography	Self-collected oncogenic papillomavirus testing	Mailed invitation involving attending physician	Increasing fee for performance concerning UCC screening
Women receiving free supplementary universal health coverage	Current stakeholders and state-registered nurses and radiologists during mammography		Mailing of screening prescription	Communication of lists of unscreened women to practitioner
Women over 50 years old			Delivery of screening prescription by occupational physicians	Fixed fee for time spent on screening
Women from deprived areas			Delivery of screening prescription by student health services	Remuneration of consultations dedicated to uterine cervical cancer screening

^a^ This first ‘neutral’ modality reflected UCC (Uterine Cervical Cancer) screening opportunistic program status of French women between 25 and 65 years old

The scenarios were each composed of five attributes corresponding to five ways of enhancing participation in UCC screening in order to promote the principle of proportionate universalism and GPs’ involvement. Two samples of invited practitioners were constituted using the same forms in the last trimester of 2014: a postal survey of 250 GPs and 250 gynaecologists working in French regions with no pilot UCC screening programme, randomly selected from the national database of the French National Medical Council using a simple random sampling procedure, and an online survey mailed to all 2594 GPs and 90 gynaecologists listed in the database of the regional union of private practitioners in the French region Midi-Pyrénées, which does not have any experimental organized UCC screening programme.

Only complete questionnaires received before April 1, 2015 were included in the analysis. A random effect probit model was applied to account for the correlation between the 11 pairwise choices made by the same physician. The model based on the resulting additive linear utility function was as follows:

$$ \Delta {{\mathrm{U}}_{\mathrm{i}}}^{\mathrm{A}\ \mathrm{vs}\ \mathrm{B}}={\upbeta}_0+{\upbeta}_1\ast {\mathrm{TAR}}^{\mathrm{A}\ \mathrm{vs}\ \mathrm{B}}+{\upbeta}_2\ast {\mathrm{A}\mathrm{CT}}^{\mathrm{A}\ \mathrm{vs}\ \mathrm{B}}+{\upbeta}_3\ast {\mathrm{TEC}}^{\mathrm{A}\ \mathrm{vs}\ \mathrm{B}}+{\upbeta}_4\ast {\mathrm{IFE}}^{\mathrm{A}\ \mathrm{vs}\ \mathrm{B}}+{\upbeta}_5\ast {\mathrm{IMG}}^{\mathrm{A}\ \mathrm{vs}\ \mathrm{B}}+{\nu}_{\mathrm{i}} $$

where TAR refers to women targeted respectively by scenario A and scenario B, ACT are the potential new stakeholders in the UCC screening, TEC are the new UCC screening techniques, IFE is the inducement to women, and IMG is the inducement to GPs. The term β_0_ is an unobservable error term representing the random variation across individuals. The term ν_i_ is the marginal utility rate. The β_1_ to β_5_ parameters are marginal utility of each attribute i.e. utilities withdrawn from an additional unit of each of the characteristics of the scenario. A marginal utility close to null denotes disinterest for the proposal. A positive marginal utility (significantly greater than 0) denotes adherence to the proposal. A negative marginal utility (significantly lower than 0) denotes reluctance to the proposal. The analysis was stratified according to medical speciality of respondents (GPs or gynaecologists), since they both are involved in the gynaecological care of women in France with potentially different interest. Results were considered as statistically significant when *p* <  0.05. The covariates tested were based on a priori knowledge. All the models were run on R software version 3.2.2 (R Core Team, 2015). Mandatory declarations were made to the ethics committee (*CPP Nord-Ouest III)* and the CNIL, which is the French Data Protection Authority.

## Results

Table 2 presents the characteristics of the 123 respondents included in the analysis. The response rate was low (15.6% for postal survey, and 2.8% for online survey where all questions had to be answered). Of the 162 respondents only 123 were included after excluding incomplete responses regarding scenario choices or medical speciality. Among the included respondents, 88 practitioners were GPs and 35 were gynaecologists. There was an equal proportion of men and women among the gynaecologists versus two thirds of women among the GPs. The mean ages were 59.3 years old (standard deviation (SD) = 9.0 years old) for gynaecologists and 55.8 years old (SD = 1.2 years old) for GPs.

**Table 2:** Characteristics of respondents

	General practitioners (*N* = 88)	Gynaecologists^a^ (*N* = 35)	***p***-value
	N (%)	N (%)	
**Gender**			
Man	31 (35.2)	17 (50.0)	0.20^†^
Woman	57 (64.8)	17 (50.0)	
**Age:** mean (SD^b^)	55.8 (9.0)	59.3 (1.2)	
**Geographical area of practice**			
Urban	23 (26.1)	20 (58.8)	0.0008^†^
Semi-rural	43 (48.9)	13 (38.2)	
Rural	22 (25.0)	1 (2.9)	
**Fixed practice**			
Yes	85 (96.6)	32 (94.1)	
No	3 (3.4)	2 (5.98)	
**Duration (yrs) of fixed practice:** mean (SD^b^)	21.4 (10.5)	25.5 (0.7)	
**Number of patients seen per day**			
Up to 15 patients	9 (10.2)	5 (14.7)	
Between 16 and 25 patients	53 (60.2)	22 (64.7)	
Over 25 patients	26 (29.5)	7 (20.6)	
**UCC**^c^**screening by cervical smear**			
Oneself	67 (76.1)	34 (100.0)	
Medical laboratory	9 (10.2)		
Gynaecologists	12 (13.6)		
**Type of smear practised**			
Pap smear	22 (31.9)	6 (17.6)	0.19^†^
Liquid-based cytology	34 (49.3)	23 (67.6)	
According to each sample	13 (18.8)	5 (14.7)	

^a^ One of the gynaecologists responded only to the pairwise choices, leading to one missing value for each variable in this table for these practitioners

^b^ Standard Deviation

^**c**^ Uterine Cervical Cancer

^†^ Overall chi-square test

In Table 3, negative coefficients reveal reluctance regarding the proposed item, whilst positive coefficients reveal adhesion. Adding additional actions targeting women from areas with low rates of screening or unscreened women was advocated by gynaecologists and GPs for the former, and only by GPs for the latter, whereas targeting of women 50 years old or those living in disadvantaged areas were rejected by both categories of physicians. Using self-collected oncogenic HPV as a routine screening test was rejected by both gynaecologists and GPs. Extending the panel of Pap smear stakeholders was advocated by GPs but not by gynaecologists. However, GPs did not favour extending the panel of Pap smear prescribers to student health services or occupational physicians. They were in favour of inducements to GPs to promote screening. This would consist in providing them with lists of unscreened women among patients of whom they are the declared attending physician, on the one hand, and sending invitations by letter to their patients provided that their identity is shown in the letter. The latter proposal was also advocated by gynaecologists. Finally, GPs advocated the inducement of a fixed remuneration for time spent on screening consultations.

**Table 3:** Random effect probit models concerning general practitioners and gynaecologists separately (only significant coefficients shown)

	General practitioners (*n* = 88 subjects leading to 1936 responses)***				Gynaecologists (*n* = 35 subjects leading to 770 responses)***			
**Attribute** - Modalities	Coefficients	S.E. **	(95% CI)^†^	*p*-value	Coefficients	S.E. **	(95% CI)^†^	*p*-value
(Intercept)	−0.10			NS*	0.03			NS*
**Population of women targeted**				< 0.01				< 0.01
- Unscreened	0.52	0.13	(0.27; 0.76)	< 0.01				NS*
- Women over 50 years old	−0.47	0.13	(−0.73; −0.22)	< 0.01	− 0.51	0.21	(− 0.92; − 0.10)	0.01
- From deprived areas	− 0.33	0.12	(-0.58; -0.09)	< 0.01	−0.61	0.20	(-0.99; -0.24)	< 0.01
- From areas with low rates of screening	0.33	0.14	(0.05; 0.60)	0.02	0.70	0.23	(0.25; 1.15)	< 0.01
- Receiving free supplementary universal health care				NS*				NS*
**Stakeholders in act of screening**				< 0.01				< 0.01
- Current stakeholders and state-registered nurses	0.44	0.10	(0.24; 0.63)	< 0.01				NS*
- Current stakeholders and state-registered nurses and radiologists during mammography	0.30	0.09	(0.12; 0.48)	< 0.01	−0.38	0.14	(− 0.65; − 0.10)	< 0.01
- Current stakeholders and radiologists during mammography				NS*				NS*
**Technique(s) of uterine cervical cancer screening**				< 0.01				< 0.01
- Self-collected oncogenic papillomavirus testing	−0.61	0.09	(− 0.78; − 0.44)	< 0.01	− 0.45	0.14	(− 0.71; − 0.18)	0.01
- Choice between pap smear or self-collected oncogenic papillomavirus testing	− 0.16	0.08	(− 0.32; −4e^−3^)	< 0.05				NS*
**Inducement to women to perform screening**				< 0.01				NS*
- Mailed invitation involving attending physician	0.44	0.11	(0.22; 0.66)	< 0.01				
- Mailed invitation without involving attending physician	−0.42	0.12	(−0.66; − 0.19)	< 0.01				
- Delivery of screening prescription by student health services	−0.32	0.13	(-0.59; -0.06)	0.02				
- Mailing of screening prescription				NS*				
- Delivery of screening prescription by occupational physicians				NS*				
**Inducement to general practitioners**				< 0.01				0.01
- Communication of lists of unscreened women to their practitioner	0.71	0.12	(0.48; 0.94)	< 0.01	0.37	0.18	(0.02; 0.72)	0.04
- Fixed fee for time spent on screening	0.42	0.12	(0.17; 0.66)	< 0.01				NS*
- Increasing fee for Pap smear				NS*				NS*
- Increasing fee for performance concerning uterine cervical cancer screening				NS*				NS*
- Remuneration of consultations dedicated to uterine cervical cancer screening				NS*				NS*

* *NS:* Not Significant

** *S.E*: Standard Error

*** n is number of observations in model and not the number of practitioners whose responses could be included (i.e. without any missing data), which is n/22

^†^ 95% CI: 95% confidence interval

## Discussion

Among the options proposed for reducing uptake inequalities in UCC screening, GPs favoured those resulting in their greater involvement in UCC screening, particularly the idea of a list of followed unscreened women being sent to them. However, both GPs and gynaecologists rejected oncogenic HPV self-sampling as a primary screening method. Both agreed about the need to target women from areas with low rates of screening and more generally unscreened women (for GPs only), but tended to reject the targeting of women over 50 years old or those from deprived areas.

The results of this DCE study should be viewed with caution, considering the very low participation rate in both the national postal survey and the regional online survey. This could be due to the topic (UCC screening for GPs since it is still mostly performed by gynaecologists, health inequalities for both types of physicians), the complexity and length of the survey or the DCE method used (around 17.0% of respondents returned a form in which not all of the 11 choices requested were given), or to the over-solicitation of practitioners by research surveys. Although the 11 binary choices in the DCE provided a countervailing statistical power in the model, the small number of analysable questionnaires severely restricted our findings (several modalities revealing no significant opinion in any direction) and the representativeness of the results.

However, the physicians’ preferences suggested by our results raise several issues regarding the implementation of organized UCC screening adopted in France since the survey was made. Among the options proposed for reducing uptake inequalities in UCC screening, GPs favoured those resulting in their greater involvement in it, particularly the idea of a list of followed unscreened women being sent to them. However, both GPs and gynaecologists rejected oncogenic HPV self-sampling as a primary screening method. Both agreed about the need to target women from areas with low rates of screening and more generally unscreened women (for GPs only) but tended to reject the targeting of women over 50 years old or those from deprived areas.

The rejection of some innovative UCC screening modalities raises the issue of resistance to change in GPs [37]. Their agreement about the need to target non-screened women particularly confirmed the need they felt to increase women’s uptake of screening [2, 18]. The current national implementation of organized UCC screening is based on applying this principle and will probably receive the support of practitioners.

The rejection of targeting women over 50 years old for screening or those from disadvantaged areas where uptake is lower may be interpreted in several ways. On one hand, it could be because physicians whose practice is dedicated to individuals find it difficult to grasp the importance of sub-groups with low participation at a population level, combined with a natural tendency to focus unconsciously only on their own patients. On the other hand, it could reflect the difficulty they experience in adopting the principle of proportionate universalism that is increasingly being propounded in Europe in the fight against health inequalities [32]. According to this principle, health care is available for all citizens, but the intensity of action is proportionate to the needs. Further studies are needed to confirm the hypothesis of the reluctance of physicians to adhere to this principle. If confirmed, we would need to understand its scope and whether it is limited to preventive actions, and its rationale (fear of stigmatization of people; fear of infringement of the patient’s freedom of choice; attachment to equality and autonomy; fear of ineffectiveness, inefficiency or even paradoxical counterproductive effects [34, 36, 38] …). After initial implementation in its current published structured, a second phase of applying the principle of proportionate universalism in UCC organized screening has already been recommended [39, 40]. The adhesion of practitioners to it will have to be sought before its implementation since they will be called upon to play a role in sampling.

UCC screening by oncogenic HPV research self-sampling did not meet the favour of either the gynaecologists or the GPs. This finding is not in agreement with the literature about the acceptance level of GPs [41]. Several hypotheses could explain this discrepancy. First, screening by HPV self-sampling might have been considered as a complete self-screening strategy, rather than a two-step screening procedure involving classical Pap smear in the event of a positive test. Second, the contribution of HPV self-sampling in terms of risk stratification and its role in decision-making in gradual UCC screening [42] for unscreened refractory women was not detailed in the questionnaire, so their proposal in the scenarios could also have been misunderstood. Nevertheless, this method is often preferred by women refractory to screening [6–8, 43], especially when it is presented as a community issue [6, 38]. French GPs are currently involved little in UCC screening in France, although they could play an important role in the prevention and management of cancers including UCC [33]. Given the other preferences expressed in our study, the practitioners were probably expressing the desire to be centrally involved in organized screening campaigns, as is the case in other countries [33, 35, 44, 45]. However, the French health authorities have recently published recommendations for incorporating these tests into the cancer screening strategy [46]. Nevertheless, the recommendations mentioned both sampling methods: by a professional or by self-collection. The implementation of these recommendations will therefore need to anticipate the potential collateral effects on screening by all parties.

In view of the current findings, two complementary studies should now be conducted to accompany the national organized screening programme. A qualitative study should explore the difficulties that GPs expressed with respect to the principle of proportionate universalism and self-collected oncogenic HPV testing that the authorities and various studies have propounded to overcome the limitations of interventions aimed at reducing inequalities. In addition, the implementation of organized UCC screening in France should be accompanied by a political awareness of the socio-economic inequalities that such changes may engender.

## Conclusions

Gynaecologists and especially GPs were in favour of the greater involvement of the latter in organized UCC screening in order to reduce screening inequalities. However, they refused any application of the principle of proportionate universalism, especially targeting deprived women, and self-collected oncogenic HPV testing. Their refusal should be confirmed and explored before implementing these screening modalities in the national organized UCC screening programme in order to achieve a reduction in health care inequalities. Exploring women’s perspectives (most and least deprived women) should also be considered in future research agenda.

## Supplementary information

**Additional file 1.** Blank English language copy of questionnaire

## Data Availability

The datasets used and/or analysed during the current study are available from the corresponding author on reasonable request.

## Abbreviations

CRC: Colorectal cancer
GPs: General practitioners
HIS: Health insurance schemes
HPV: Oncogenic human papillomavirus
UCC: Uterine cervical cancer
