# Supplementary material for: Social inequalities in cervical cancer screening: a discrete choice experiment among French general practitioners and gynaecologists
Source: BMC Health Serv Res. 2020 Jul 27;20:693. doi: 10.1186/s12913-020-05479-w (PMC7385880; doi:10.1186/s12913-020-05479-w)
Supplement: Supplementary file 1 — Additional file 1. Blank English language copy of questionnaire [file 12913_2020_5479_MOESM1_ESM.docx]

**Additional file**

**Additional file:** blank English language copy of questionnaire


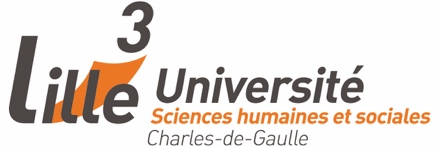

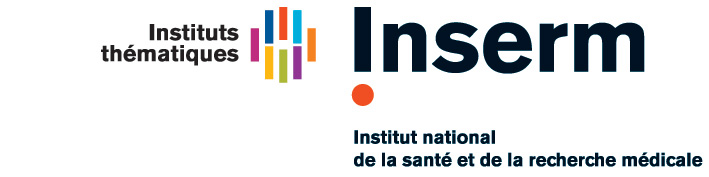

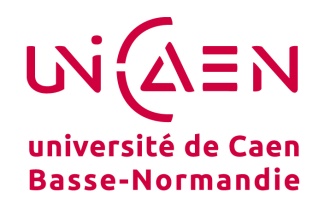


**REMEDE Project**

**Questionnaire for general practitioners / gynaecologists**

Le XX XXXXXXXXX 2014

Mrs, Miss, Mr,

Doctor,

We are inviting you to become involved in the REMEDE research project. The aim of this project is to evaluate the acceptability of different scenarios to reduce inequality in cervical cancer screening participation.

Accordingly, we would be pleased if you accepted to answer this [online] questionnaire about cervical smears in cervical cancer screening, which should take you about 15 to 20 minutes.

The questionnaire will invite you 11 times to express your preference for a scenario among proposed scenarios that include vary items relating to:

- - the population of women targeted by the scenario,
  - the stakeholders (in the act of screening itself),
  - the technique(s) of uterine cervical cancer screening,
  - the inducement to women to undergo screening,
  - the inducement to general practitioners (in order to have women screened).

The methodology of choices may seem unusual, but it is a well-proven and scientifically validated method that we are using here.

We thank you in advance for your participation and we remain at your disposal for any further information.

Respectfully,

Doctor Thibaut RAGINEL.

**PREFERENCES ABOUT CONCEIVED SCENARIOS**

When planning measures to reduce cervical cancer screening uptake inequalities, and without considering changing the recommendations about screening frequency: for each pair of scenarios, please pick the scenario you prefer to reduce cervical cancer screening uptake inequalities. It’s really important that you pick an answer for all pairs of scenarios.

Considering the scenarios, targeting a population of women in any scenario means targeting them in the whole scenario (therefore this includes all other items in this scenario). The term “smear” refers only to Pap smear; the term "self-collected oncogenic papillomavirus testing" refers to a vaginal sample to test for the presence of an oncogenic papillomavirus that can be taken by the woman herself; the term “screening” may refer to either of these two screening methods.

For example, for the following hypothetic scenario, you should read it as follows:

|  | SCENARIO X |
| --- | --- |
| Population of  targeted women | Women over 50 years old |
| Stakeholders (in the act of  screening itself) | Current stakeholders^1^ and state-registered nurses |
| Technique(s) of uterine  cervical cancer screening | Self-collected oncogenic papillomavirus testing |
| Inducement to women  to undergo screening | Delivery of screening prescription by occupational physicians |
| Inducement to  general practitioners | Increasing fee for performing Pap smear |

Without considering changing any other aspect of current screening modalities, this scenario concerns only women over 50 years old. In this scenario, they may be screened by current stakeholders and licensed nurses who would offer them a self-collected oncogenic papillomavirus test. In order to induce women over 50 to undergo screening, occupational physicians would give them a screening prescription, i.e. a prescription for a self-collected oncogenic papillomavirus test. In order to induce general practitioners to have women over 50 years old screened, their fee for performing a Pap smear with these specific women would be increased.

| CHOICE 1 | SCENARIO A | SCENARIO B |
| --- | --- | --- |
| Population of  targeted women | Women receiving free supplementary universal health coverage | Women over 50 years old |
| Stakeholders (in the act of  screening itself) | Current stakeholders^1^ and state-registered nurses | Current stakeholders^1^ and radiologists during mammography |
| Technique(s) of uterine  cervical cancer screening | Choice between Pap smear or self-collected oncogenic papillomavirus testing | Self-collected oncogenic papillomavirus testing |
| Inducement to women  to undergo screening | Mailed invitation involving attending physician | Delivery of screening prescription by occupational physicians |
| Inducement to  general practitioners | Increasing fee for performing Pap smear | Fixed fee for time spent on screening |

1 Current stakeholders: gynaecologists, obstetricians-gynaecologists, cytopathology or medical biology laboratory, general practitioners, midwives

Which scenario would you prefer for reducing the differences in participation in screening for cervical cancer?

(Tick the box corresponding to your choice) ☐ A ☐ B

| CHOICE 2 | SCENARIO A | SCENARIO B |
| --- | --- | --- |
| Population of  targeted women | Women from areas with low rates of screening | Women over 50 years old |
| Stakeholders (in the act of  screening itself) | Current stakeholders^1^ and radiologists during mammography | Current stakeholders^1^ and state-registered nurses and radiologists during mammography |
| Technique(s) of uterine  cervical cancer screening | Choice between Pap smear or self-collected oncogenic papillomavirus testing | Self-collected oncogenic papillomavirus testing |
| Inducement to women  to undergo screening | Mailing of screening prescription | Mailed invitation involving attending physician |
| Inducement to  general practitioners | Increasing fee for performing Pap smear | Remuneration of consultations dedicated to uterine cervical cancer screening |

1 Current stakeholders: gynaecologists, obstetricians-gynaecologists, cytopathology or medical biology laboratory, general practitioners, midwives

Which scenario would you prefer for reducing the differences in participation in screening for cervical cancer?

(Tick the box corresponding to your choice) ☐ A ☐ B

| CHOICE 3 | SCENARIO A | SCENARIO B |
| --- | --- | --- |
| Population of  targeted women | Unscreened women | Women receiving free supplementary universal health coverage |
| Stakeholders (in the act of  screening itself) | Current stakeholders^1^ and state-registered nurses | Current stakeholders^1^ and state-registered nurses and radiologists during mammography |
| Technique(s) of uterine  cervical cancer screening | Pap smear | Self-collected oncogenic papillomavirus testing |
| Inducement to women  to undergo screening | Mailing of screening prescription | Mailed invitation without involving attending physician |
| Inducement to  general practitioners | Remuneration of consultations dedicated to uterine cervical cancer screening | Increasing fee for performance concerning uterine cervical cancer screening |

1 Current stakeholders: gynaecologists, obstetricians-gynaecologists, cytopathology or medical biology laboratory, general practitioners, midwives

Which scenario would you prefer for reducing the differences in participation in screening for cervical cancer?

(Tick the box corresponding to your choice) ☐ A ☐ B

| CHOICE 4 | SCENARIO A | SCENARIO B |
| --- | --- | --- |
| Population of  targeted women | Unscreened women | Women from deprived areas |
| Stakeholders (in the act of  screening itself) | Current stakeholders^1^ and radiologists during mammography | Current stakeholders^1^ |
| Technique(s) of uterine  cervical cancer screening | Pap smear | Choice between Pap smear or self-collected oncogenic papillomavirus testing |
| Inducement to women  to undergo screening | Mailed invitation involving attending physician | Mailed invitation without involving attending physician |
| Inducement to  general practitioners | Increasing fee for performance concerning uterine cervical cancer screening | Fixed fee for time spent on screening |

1 Current stakeholders: gynaecologists, obstetricians-gynaecologists, cytopathology or medical biology laboratory, general practitioners, midwives

Which scenario would you prefer for reducing the differences in participation in screening for cervical cancer?

(Tick the box corresponding to your choice) ☐ A ☐ B

| CHOICE 5 | SCENARIO A | SCENARIO B |
| --- | --- | --- |
| Population of  targeted women | Women receiving free supplementary universal health coverage | Women over 50 years old |
| Stakeholders (in the act of  screening itself) | Current stakeholders^1^ | Current stakeholders^1^ and state-registered nurses |
| Technique(s) of uterine  cervical cancer screening | Pap smear | Choice between Pap smear or self-collected oncogenic papillomavirus testing |
| Inducement to women  to undergo screening | Mailing of screening prescription | Current incentives to screening |
| Inducement to  general practitioners | Communication of lists of unscreened women to practitioner | Increasing fee for performance concerning uterine cervical cancer screening |

1 Current stakeholders: gynaecologists, obstetricians-gynaecologists, cytopathology or medical biology laboratory, general practitioners, midwives

Which scenario would you prefer for reducing the differences in participation in screening for cervical cancer?

(Tick the box corresponding to your choice) ☐ A ☐ B

| CHOICE 6 | SCENARIO A | SCENARIO B |
| --- | --- | --- |
| Population of  targeted women | Women from areas with low rates of screening | Women from deprived areas |
| Stakeholders (in the act of  screening itself) | Current stakeholders^1^ and state-registered nurses and radiologists during mammography | Current stakeholders^1^ and state-registered nurses |
| Technique(s) of uterine  cervical cancer screening | Pap smear | Self-collected oncogenic papillomavirus testing |
| Inducement to women  to undergo screening | Current incentives to screening | Delivery of screening prescription by student health services |
| Inducement to  general practitioners | Remuneration of consultations dedicated to uterine cervical cancer screening | No change in remuneration or logistic support |

1 Current stakeholders: gynaecologists, obstetricians-gynaecologists, cytopathology or medical biology laboratory, general practitioners, midwives

Which scenario would you prefer for reducing the differences in participation in screening for cervical cancer?

(Tick the box corresponding to your choice) ☐ A ☐ B

| CHOICE 7 | SCENARIO A | SCENARIO B |
| --- | --- | --- |
| Population of  targeted women | Unscreened women | Women over 50 years old |
| Stakeholders (in the act of  screening itself) | Current stakeholders^1^ and state-registered nurses and radiologists during mammography | Current stakeholders^1^ and radiologists during mammography |
| Technique(s) of uterine  cervical cancer screening | Choice between Pap smear or self-collected oncogenic papillomavirus testing | Pap smear |
| Inducement to women  to undergo screening | Delivery of screening prescription by student health services | Mailed invitation without involving attending physician |
| Inducement to  general practitioners | Communication of lists of unscreened women to practitioner | No change in remuneration or logistic support |

1 Current stakeholders: gynaecologists, obstetricians-gynaecologists, cytopathology or medical biology laboratory, general practitioners, midwives

Which scenario would you prefer for reducing the differences in participation in screening for cervical cancer?

(Tick the box corresponding to your choice) ☐ A ☐ B

| CHOICE 8 | SCENARIO A | SCENARIO B |
| --- | --- | --- |
| Population of  targeted women | All women | Women from deprived areas |
| Stakeholders (in the act of  screening itself) | Current stakeholders^1^ | Current stakeholders^1^ and radiologists during mammography |
| Technique(s) of uterine  cervical cancer screening | Pap smear | Self-collected oncogenic papillomavirus testing |
| Inducement to women  to undergo screening | Delivery of screening prescription by student health services | Mailed invitation involving attending physician |
| Inducement to  general practitioners | Remuneration of consultations dedicated to uterine cervical cancer screening | Communication of lists of unscreened women to practitioner |

1 Current stakeholders: gynaecologists, obstetricians-gynaecologists, cytopathology or medical biology laboratory, general practitioners, midwives

Which scenario would you prefer for reducing the differences in participation in screening for cervical cancer?

(Tick the box corresponding to your choice) ☐ A ☐ B

| CHOICE 9 | SCENARIO A | SCENARIO B |
| --- | --- | --- |
| Population of  targeted women | All women | Unscreened women |
| Stakeholders (in the act of  screening itself) | Current stakeholders^1^ and state-registered nurses and radiologists during mammography | Current stakeholders^1^ |
| Technique(s) of uterine  cervical cancer screening | Choice between Pap smear or self-collected oncogenic papillomavirus testing | Self-collected oncogenic papillomavirus testing |
| Inducement to women  to undergo screening | Mailing of screening prescription | Current incentives to screening |
| Inducement to  general practitioners | No change in remuneration or logistic support | Increasing fee for performing Pap smear |

1 Current stakeholders: gynaecologists, obstetricians-gynaecologists, cytopathology or medical biology laboratory, general practitioners, midwives

Which scenario would you prefer for reducing the differences in participation in screening for cervical cancer?

(Tick the box corresponding to your choice) ☐ A ☐ B

| CHOICE 10 | SCENARIO A | SCENARIO B |
| --- | --- | --- |
| Population of  targeted women | All women | Women from deprived areas |
| Stakeholders (in the act of  screening itself) | Current stakeholders^1^ and state-registered nurses | Current stakeholders^1^ and state-registered nurses and radiologists during mammography |
| Technique(s) of uterine  cervical cancer screening | Self-collected oncogenic papillomavirus testing | Pap smear |
| Inducement to women  to undergo screening | Mailed invitation without involving attending physician | Delivery of screening prescription by occupational physicians |
| Inducement to  general practitioners | Communication of lists of unscreened women to practitioner | Increasing fee for performing Pap smear |

1 Current stakeholders: gynaecologists, obstetricians-gynaecologists, cytopathology or medical biology laboratory, general practitioners, midwives

Which scenario would you prefer for reducing the differences in participation in screening for cervical cancer?

(Tick the box corresponding to your choice) ☐ A ☐ B

| CHOICE 11 | SCENARIO A | SCENARIO B |
| --- | --- | --- |
| Population of  targeted women | Women from areas with low rates of screening | Women receiving free supplementary universal health coverage |
| Stakeholders (in the act of  screening itself) | Current stakeholders^1^ | Current stakeholders^1^ and radiologists during mammography |
| Technique(s) of uterine  cervical cancer screening | Choice between Pap smear or self-collected oncogenic papillomavirus testing | Choice between Pap smear or self-collected oncogenic papillomavirus testing |
| Inducement to women  to undergo screening | Delivery of screening prescription by occupational physicians | Current incentives to screening |
| Inducement to  general practitioners | No change in remuneration or logistic support | Remuneration of consultations dedicated to uterine cervical cancer screening |

1 Current stakeholders: gynaecologists, obstetricians-gynaecologists, cytopathology or medical biology laboratory, general practitioners, midwives

Which scenario would you prefer for reducing the differences in participation in screening for cervical cancer?

(Tick the box corresponding to your choice) ☐ A ☐ B

**SOME QUESTIONS TO FINISH**

In your opinion, regarding cervical cancer screening, complete the following table:

(please provide one answer per line **to both questions if applicable**)

|  | do you think there are differences in women's participation according to: | | **If you do**, do you think that interventions should be considered to reduce these differences in participation according to: | |
| --- | --- | --- | --- | --- |
|  | Yes | No | Yes | No |
| the age of the woman |  |  |  |  |
| the location of the woman |  |  |  |  |
| the socio-economic situation of the woman's home |  |  |  |  |
| the woman’s perception of the usefulness of this screening |  |  |  |  |
| the physician’s individual perception of the usefulness of this screening |  |  |  |  |

- If you were allowed to book time for appointments dedicated to prevention and paid on a fixed fee basis: Would you have time for this type of consultation? ☐ Yes ☐ No
- Would you offer these appointments? ☐ Yes ☐ No
- If you would, at what hourly rate (tick only one box):

☐ 50 € / hour

☐ 100 € / hour

☐ 150 € / hour

☐ 200 € / hour

**DEMOGRAPHIC AND PRACTICE DATA OF GENERAL PRACTITIONERS**

Gender: ☐ Man ☐ Woman

Age: ……….………… years old

Number of department where practicing: ……….……………......

Practice modality: (tick only one box)

☐ private practice ☐ mixed practice

Registered sector of private practice: (tick only one box)

☐ registered sector 1

☐ registered sector 2

☐ unregistered

Geographical location of main practice: (tick only one box)

☐ rural practice

☐ semi-rural practice

☐ urban practice

Registered practice: (tick only one box) ☐ Yes ☐ No

If registered, time since registration: ……….………… years

When at the office, number of patients seen in consultation per day: (tick only one box)

☐ 15 patients or fewer

☐ 16 to 25 patients

☐ more than 25 patients

About your habits related to cervical cancer screening: (tick only one box)

☐ You do the gynaecological follow-up of the patients requesting it and you perform the cervical smears yourself

☐ You do the gynaecological follow-up of the patients requesting it but you prescribe the cervical smears to be done at the laboratory

☐ Most patients are followed up by a gynaecologist but you would be amenable to doing it yourself

☐ You do not do any gynaecological follow-up and you systematically refer women to a gynaecologist

If you perform cervical smears yourself, do you do: (tick only one box)

☐ Conventional Pap smears

☐ Liquid-based cytology

☐ Either conventional Pap smears or liquid-based cytology

**DEMOGRAPHIC AND PRACTICE DATA OF GYNAECOLOGISTS**

Gender: ☐ Man ☐ Woman

Age: ……….………… years old

Number of department where practicing: ……….……………......

Practice modality: (tick only one box)

☐ private practice ☐ mixed practice

Registered sector of private practice: (tick only one box)

☐ registered sector 1

☐ registered sector 2

☐ unregistered

Geographical location of main practice: (tick only one box)

☐ rural practice

☐ semi-rural practice

☐ urban practice

Registered practice: (tick only one box) ☐ Yes ☐ No

If registered, duration since registration: ……….………… years

When at the office, number of patients seen in consultation per day: (tick only one box)

☐ 15 patients or fewer

☐ 16 to 25 patients

☐ more than 25 patients

Do you regularly prescribe uterine cervical cancer screening? (tick only one box)

☐ Yes and you perform the cervical smears yourself

☐ Yes and you prescribe the cervical smears to be done at the laboratory

☐ No, the women have their cervical smears performed by their general practitioners or another gynaecologist

If you perform cervical smears yourself, do you practice: (tick only one box)

☐ Conventional” Pap smears

☐ Liquid-based cytology

☐ Either conventional Pap smears or liquid-based cytology
